# Telehealth services for global emergencies: implications for COVID-19: a scoping review based on current evidence

**DOI:** 10.1186/s12913-023-09584-4

**Published:** 2023-06-01

**Authors:** Jonathan Kissi, Caleb Annobil, Nathan Kumasenu Mensah, Joseph Owusu-Marfo, Ernest Osei, Zenobia Wooduwa Asmah

**Affiliations:** 1 School of Allied Health Sciences, Department of Health Information Management. University Post Office, University of Cape Coast, Cape Coast, Ghana; 2 Department of Epidemiology, Biostatistics and Disease Control, University for Development Studies, Tamale, Ghana; 3 Faculty of Health and Allied Health, Department of Public Health, Catholic University College of Ghana, Sunyani, Ghana

**Keywords:** High-income and low-and-middle-income countries, Pandemics, COVID-19, Telehealth services, Telecommunications

## Abstract

**Introduction:**

The availability of low-cost computing and digital telecommunication in the 1980s made telehealth practicable. Telehealth has the capacity to improve healthcare access and outcomes for patients while reducing healthcare costs across a wide range of health conditions and situations.

**Objective:**

This study compares the adoption, advantages, and challenges of telehealth services between high-income (HICs) and low-and-middle-income countries (LMICs) before and during the COVID-19 pandemic.

**Methods:**

Preferred Reporting Items for Systematic Reviews and Meta-Analyses (PRISMA) guidelines were followed. The key search terms were: “Telehealth”, “Telehealth in HICs”, “Telehealth in LMICs”, “Telehealth before COVID-19”, “Telehealth during COVID-19”. We searched exhaustively ProQuest, Scopus, Web of Science, Google Scholar, CINAHL, and EMBASE databases from 2012. Booleans OR/AND were combined with key search terms to increase relevant search results. The literature search and selection process followed the Sample, Phenomena of Interest, Design, Evaluation, and Research (SPIDER) question format.

**Results:**

The adoption of telehealth before COVID-19 was generally low in both HICs and LMICs. The impact of COVID-19 accelerated the adoption of telehealth at the facility level but not nationwide in both high-income countries and LMICs. The rapid adoption of telehealth at the facility level in both high-income and LMICs introduced several challenges that are unique to each country and need to be addressed.

**Conclusion:**

The lack of national policies and regulations is making the adoption of telehealth at the national level challenging in both high and low-middle-income countries. Governments and Stakeholders of healthcare must consider telehealth as a healthcare procedure that should be deployed in clinical working procedures. Primary quantitative and qualitative studies must be conducted to address challenges encountered during the pilot implementation of telehealth services in both high-income countries and LMICs before and during pandemics.

## Introduction

The availability of low-cost computing and digital telecommunication in the 1980s made telehealth practicable [1]. Telehealth is said to have gone through three generations. The first generation was reactive telehealth systems which focused mainly on social alarms. The second generation was proactive telehealth systems that automated responses based on sensor information. And the third generation is an integrated telehealth system that uses virtual communities to enhance patients’ quality of life [2]. Telehealth may be defined as the use of electronic media to assist a broad range of remote services, such as patient care, education, and monitoring [3]. Telehealth has the capacity to improve healthcare access and outcomes for patients while reducing healthcare costs across a wide range of health conditions and situations [4]. Telehealth is perceived as the mitigator of healthcare provider shortages and remote access to health services [5]. This addresses the point that telehealth presents an opportunity to improve Universal Health Coverage (UHC) [6].

It is to this effect that this study compares the adoption of telehealth between high-income countries and LMICs before and during the COVID-19 pandemic. This study also assesses the advantages and challenges of telehealth before and during the COVID-19 pandemic in the context of high-income countries and LMICs.

## Methodology

### Literature search

**Fig. 1:**
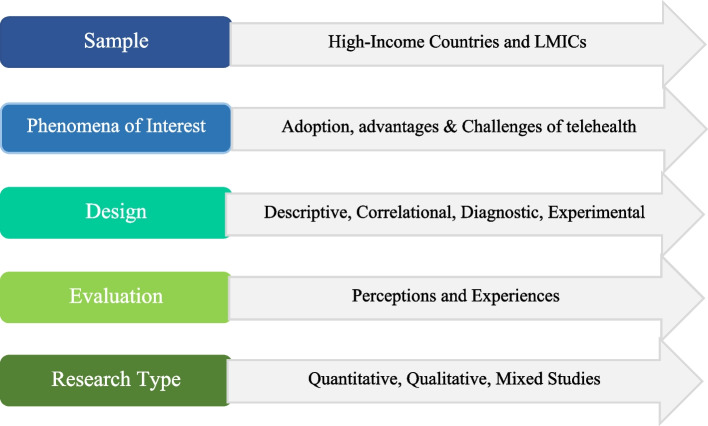
SPIDER question format used for study analysis

Studies were downloaded from six databases, namely: ProQuest, Scopus, Web of Science, Google Scholar, CINAHL (Cumulative Index to Nursing and Allied Health Literature), and EMBASE. In selecting the included studies for this paper, Preferred Reporting Items for Systematic Reviews and Meta-Analyses (PRISMA) guidelines were followed. The key search terms were: “Telehealth”, “Telehealth in High-Income Countries”, “Telehealth in LMICs”, “Telehealth before COVID-19”, “Telehealth during COVID-19”. Booleans OR/AND were combined with key search terms to increase relevant search results. The literature search and selection process followed the SPIDER question format (see Fig. 1). Three reviewers (who are also coauthors) independently checked the title/abstracts of all the listed studies for inclusion. The full texts of potentially qualifying studies were retrieved for detailed assessment using a scoping review. An author separately tested the eligibility of potentially qualifying studies.

### Inclusion and exclusion criteria

The eligibility criterion were the study must be published in the English language. Studies published before 2012 were excluded from this study. Grey literature, dissertations, and unpublished studies were excluded from this study. Only peer-reviewed published studies with high indexing were included. Studies that addressed the title, key search terms, and objectives of the study were included.

Included studies were first classified into high-income countries and LMICs. The classification of these studies into high-income countries and LMICs was to determine if financial disparity played a role in the penetration of telehealth before COVID-19. If yes, did the COVID-19 pandemic accelerate the use of telehealth and how was it possible in financial constraint countries? Studies conducted in high income countries were categorized under “high-income countries” while studies conducted in lower-and-middle-income countries were categorized under LMICs. The World Bank classification of countries was used for this study.

## Results

**Fig. 2:**
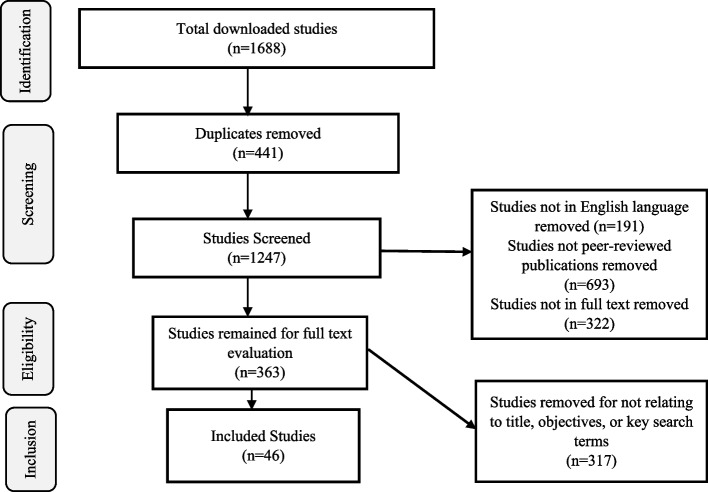
PRISMA flow chart showing literature search and selection of studies

A total of 1688 studies were downloaded from the databases mentioned under section 2.1. A total of 441 duplicates were removed using Mendeley referencing software. A total of 191 studies not written in English language were removed. 371 studies were not peer-reviewed publications, hence, removed. A total of 322 studies were removed for not being full-text articles. A total of 363 studies remained for full-text review. Post-full-text review, a total of 317 studies were removed for not relating to the study title or objectives, or key search terms. A total of 46 studies remained and were included in this study. Figure 2 (PRISMA chart) summaries the results of this study.

The classified studies were further grouped into “before COVID-19 pandemic” and “during COVID-19 pandemic”. The grouped studies were placed under the subject matter they addressed. Table 1 shows the distribution of included studies based on the subject matter they addressed. Pre COVID-19 pandemic, some high-income countries like Portugal were adopting telehealth as a complementary healthcare service that only compensated for existing asymmetries and inadequate resources [7] (see Table 2). Other high-income countries like Australia, the United States of America, the United Kingdom, Canada, and Brazil were using telehealth systems to provide specialised services for people living in remote communities who would have traveled long distances to access specialised services [4, 10,8-] (see Table 3). As postulated by Van Dyk L [11], there was slow or no widespread adoption of telehealth in most low-and-middle-income countries (LMICs). Countries like Nigeria and Burkina Faso recorded slow progress with regards to telehealth adoption, and this was as a result of lack of political will [12] (see Table 4). During this COVID-19 pandemic, telehealth became a novel alternative for offering musculoskeletal physical therapy services [13] (see Table 5). However, the introduction of telehealth before and during the COVID-19 pandemic posed certain challenges for both high-income countries and LMICs.

**Table 1:** Results of included articles

	**Before COVID-19**	
	**High-Income Countries**	**LMICs**
1. Telehealth adoption	4	2
2. Application in Specialised Services & Advantages	6	5
3. Challenges	2	2
	**During COVID-19**	
1. Telehealth adoption	6	3
2. Application in Specialised Services & Advantages	5	3
3. Challenges	4	4

**Table 2:** Adoption of telehealth before COVID-19

High-Income Countries (HICs)		
Year	Authour(s)	Key Findings
2017	Kayyali et al., [14]	Although the Whole System Demonstrator (WSD) project, which is considered the world’s largest randomised controlled trial (RCT) on telehealth showed that telehealth can significantly reduce hospital admission rates (*P=*0.0017), the length of stay (*P=*0.023), and mortality rates (*P<*0.001), telehealth adoption is still poor in the UK.
2012	Zanaboni & Wootton, [1]	Almost no telehealth application had reached large-scale and enterprise-wide adoption as of 2012. The widespread use of telehealth was underdeveloped and needed strengthened new research directions.
2015	Bradford et al., [10]	A study conducted in the Queensland community in Australia makes it known that out of the 60% of participants who were aware of telehealth, only 13% had used telehealth services. This shows that although people know about telehealth, only a few people use it.
2019	Maia et al., [7]	A study conducted in Portugal shows that telehealth is a complementary healthcare service and only compensates for existing asymmetries and inadequate resources.
Low-and-Middle-Income Countries (LMICs)		
2013	Wamala & Augustine, [12]	Observations from Wamala & Augustine (2013) postulate dearth commitment and efforts to the optimise use of telehealth in Africa. Before the COVID-19 pandemic, countries like Ethiopia and South Africa recorded some progress in the adoption of telehealth, while others like Nigeria and Burkina Faso recorded slow progress as a result of lack of political will.
2014	Van Dyk L. [11]	Although telehealth has the potential to increase accessibility and quality of healthcare, there was slow or no widespread adoption of telehealth in most LMICs. In South Africa, telehealth services that were successful in the pilot phase could not be sustained.

**Table 3:** Application of telehealth in specialised services & advantages before COVID-19

High-Income Countries (HICs)		
Year	Author(s)	Key Findings
2014	Bradford et al., [15]	In Australia, telehealth is equally an effective way of treating paediatric palliative care. Virtual spaces provide an opportunity for clinicians to observe the living surroundings of patients and include these observations in diagnosing conditions.
2018	Jong et al., [8]	A study in Nunavut, Canada postulates that the implementation of telehealth reduced travel costs by 50% for patients, health professionals, or both who would have traveled to seek or render specialized healthcare services.
2014	Durland et al., [9]	Some health facilities in the United States (U.S.) are using telehealth through telephone-mediated psychosocial interventions to manage depressed medical populations confronting significant barriers to face-to-face treatment.
2018	Selzler et al., [16]	In the context of Canada, features of telehealth such as telemonitoring, teleconsultation, tele-education, and telehealth-pulmonary rehabilitation are used in the management of chronic respiratory diseases.
2013	Turner & McGee-Lenon, [2]	A study that assessed the advances of telehealth over the past 10 years in the UK showed that pre-COVID-19, telecare systems provided social connectedness to the aged. Reminders were integrated into telehealth systems to alert forgetful adults to watch their favourite television programmes.
2015	Garcia et al., [4]	A study by Gracia et al. stipulates that in 2015, telehealth reduced costs associated with health conditions for patients in U.S. and Brazil while eliminating the distance between patients and doctors. Telehealth efficiently ensured clinical data sharing, patient’s visualisation and inspection through high-definition cameras, and real-time collection of vital signs.
Low-and-Middle-Income Countries (LMICs)		
2019	Siddiquee et al., [17]	The implementation of telehealth in Nepal is addressing issues such as geographical remoteness (21%), shortage of healthcare service providers (11%), extreme conditions (10%), cost (9%), service quality (9%), and real-time services (8%).
2014	Bagayoko et al., [18]	The implementation of telehealth in Mali increased patient visits from 8% to 35%. Patients who utilised telehealth saved an average cost of $25 and maximum of $75 compared to those who traveled to cities for face-to-face specialised services.
2016	Chakrabarti & Shah, [19]	As cited in Chakrabarti & Shah, clinical outcomes of telepsychiatric interventions are comparable to face-to-face treatment among patients of all ages, ethnicities, cultures and diagnostic groups across diverse clinical settings.
2015	Ganapathy K. [20]	In India, although there are only 2.67% of the total neurologists and neurosurgeons living in rural communities covering a population of 84.59 million, the implementation of telemedicine has partially resolved the acute manpower shortage. Video Conferencing (VC) systems are commercially applied to conduct teleconsultation sessions for neurological patients.
2019	Sayani et al., [21]	Telehealth is improving chronic disease outcomes in LMICs while reducing cost for patients living in LMICs.

**Table 4:** Challenges of telehealth implementation before COVID-19

High-Income Countries (HICs)		
Year	Author(s)	Key Findings
2019	Alghamdi et al., [22]	A study that assessed the adherence and dropout rates of individuals with chronic obstructive pulmonary disease (COPD) in telehealth interventions showed that there is a lack of knowledge on the effectiveness of telehealth for COPD care post-implementation. This makes it difficult to assess the impact of telehealth on COPD management.
2012	Sanders et al., [23]	A qualitative study conducted in the UK shows that patients are reluctant to risk potential disruptive changes to existing face-to-face services that are highly valued. There are difficulties in recruiting health professionals for telehealth services, where recruitment difficulties are reported at 80% refusal rate.
Low-and-Middle-Income Countries (LMICs)		
2015	Scott et al., [24]	In LMICs, telehealth has still not been integrated into existing healthcare systems. Some of the reasons are: limited resources, unreliable power supply, poor internet connectivity, and high cost for the poverty stricken.
2013	Cilliers & Flowerday, [25]	A barrier to the effective implementation of a telehealth system in LMICs is the lack of awareness regarding the telehealth system. The study further elaborates that health professionals are apprehensive when using telehealth, and this contributes to less frequent usage.

**Table 5:** Adoption of telehealth during COVID-19

High-Income Countries (HICs)		
Year	Author(s)	Key Findings
2021	James et al., [26]	The disruptive impact of COVID-19 has rapidly progressed the implementation and use of telehealth in Australian PHC as has occurred in other developed countries.
2022	Tang & Reddy, [27]	As cited in Tang & Reddy (2022), the U.S. saw an increase of 154% in telehealth visits at the end of March 2022 compared to the same period in 2019.
2021	Heneghan et al., [13]	A mixed-method study conducted in the UK and Canada postulates that although patient’s satisfaction with telehealth in musculoskeletal physical therapy is widely reported as high as face-to-face care, widespread adoption of telehealth within physical therapy has been slow.
2021	Reisinger-Kindle et al., [28]	There were 698 (39%) telehealth visits out of a total of 1788 prenatal visits by 558 patients. This shows that there was high adoption of telehealth during COVID-19 in Springfield, Massachusetts.
2022	Alpert et al., [29]	Telemedicine was not utilized in cancer care before COVID-19. The COVID-19 pandemic forced health systems to quickly adapt to telehealth use for cancer treatment.
2021	Rangachari et al., [3]	While specialties like psychiatry, cardiology, and radiology are recording higher telehealth use, others like allergy-immunology, family medicine, and gastroenterology are recording lower telehealth use.
Low-and-Middle-Income Countries (LMICs)		
2021	Cruz et al., [30]	Based on responses from a study conducted in Mozambique, 69% of respondents were willing to use teleconsultation public health services for mild illness and review consultations. It was realised that respondents were willing to adopt to telehealth use due to its relatively cheaper price of services.
2021	Ranjbar et al., [31]	Out of a total of 523 nurses and midwives who participated in the study, 73.0% had positive attitude towards telenursing and telehealth. Higher education was positively associated with the understanding of telehealth.
2020	Kazi et al., [32]	Telehealth was initially in its infancy in LMICs, however, the COVID-19 pandemic accelerated the demand for telehealth following social distancing protocols globally.

**Fig. 3:**
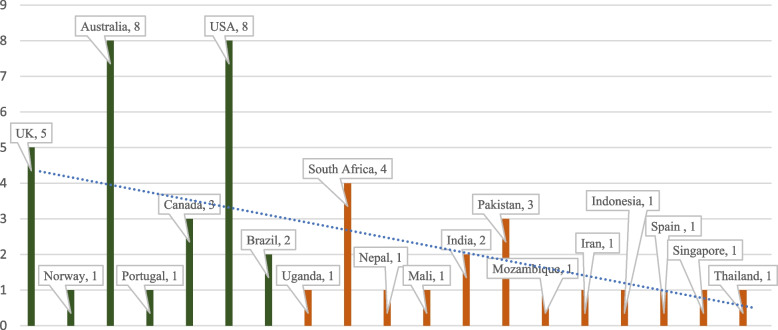
Distribution of countries based on their respective number of articles

Included studies were from 19 countries. Figure 3 shows the distribution of some studies per country and the focus of the study (focus here is with regards to high-income or LMIC). There was an exception where although a study was conducted in Spain, the focus was on LMICs.

## Discussion

### A Comparison Between High-Income Countries (HICs) and LMICs on Telehealth Before COVID-19 Pandemic

In the context of both HICs and LMICs, almost no telehealth application had reached large-scale and enterprise-wide adoption as of 2012 [1]. Before the COVID-19 pandemic, the adoption of telehealth in a high-income country like the UK was poor [14] (see Table 2). A study conducted in the Queensland community in Australia showed that out of 60% of participants who were aware of telehealth, only 13% had used telehealth services [10]. It can be inferred that although people knew about telehealth before the COVID-19 pandemic, only a few used it in certain parts of a high-income country like Australia (see Fig. 3). In Portugal, telehealth was used as a complementary healthcare service and only compensated for existing asymmetries and inadequate resources before the COVID-19 pandemic [7]. In LMICs, there was a dearth of commitment and efforts to optimise the use of telehealth (see Table 3). Before the COVID-19 pandemic, countries like Nigeria and Burkina Faso recorded slow progress in the adoption of telehealth as a result of lack of political will [12]. Van Dyk L [11] stipulates that in an LMIC like South Africa, telehealth services that were successful in the pilot phase could not be sustained (see Table 4).

In context to the application of telehealth in specialised healthcare service delivery and the advantages of telehealth, telehealth was an equally effective way of treating paediatric palliative care in a high-come country like Australia even before the COVID-19 pandemic [15] (see Fig. 3). A study in Nunavut, Canada before the COVID-19 pandemic postulates that, the implementation of telehealth reduced travel costs by 50% for patients and health professionals who would have travelled to seek or render specialised healthcare services [8]. This was similar to the post-implementation benefit of telehealth in an LMIC like Mali, where patients who patronised telehealth services saved an average cost of $25 and a maximum of $75 compared to those who traveled to cities for face-to-face specialised consultation services [18]. Telepsychiatric interventions through telephone-mediated psychosocial methods were deployed by both HICs and LMICs in managing depressed medical populations before the COVID-19 pandemic [9] (see Table 3). India, which is classified as a low-and-middle-income country commercially used video conferencing (VC) before the COVID-19 pandemic to conduct teleconsultation sessions for neurological patients [20]. Sayani et. al., [21] expounded on how telehealth improved chronic disease outcomes in LMICs. Similarly, Selzler et al., [16] elaborate on how Canada used telehealth features such as telemonitoring, teleconsultation, tele-education, and telehealth-pulmonary rehabilitation to manage chronic respiratory diseases before the COVID-19 pandemic. A study that assessed the advances of telehealth over the past 10 years in the UK showed that telehealth systems provided social connectedness to the aged. Reminders were integrated into telehealth systems to alert forgetful adults to watch their favourite television programmes [2]. While the implementation of telehealth in Nepal addressed issues of real-time services as postulated by Siddiquee et al., [17], the implementation of telehealth before the COVID-19 pandemic in the U.S. and Brazil had ensured clinical data sharing and real-time collection of vital signs (see Table 3).

There were some challenges pre and post-implementation of telehealth in both HICs and LMICs before the COVID-19 pandemic (see Table 4). A qualitative study conducted in the UK showed that patients were reluctant to risk potential disruptive changes to existing highly valued face-to-face services. In the same study, it was indicated that there are difficulties in recruiting health professionals for telehealth services, where health professionals’ refusal rate was reported at 80% [23]. A study that assessed the adherence and dropout rates of individuals with chronic obstructive pulmonary disease (COPD) in telehealth interventions showed that there is a lack of knowledge on the effectiveness of telehealth for COPD care post-implementation. This made it difficult to assess the impact of telehealth on COPD management [22]. In LMICs, telehealth had not been integrated into existing healthcare systems. Some of the reasons were: limited resources, unreliable power supply, poor internet connectivity, and high cost for the poverty-stricken [24]. A barrier to the effective implementation of a telehealth system in LMICs is the lack of awareness regarding the telehealth system [25] (see Table 4).

### A Comparison Between HICs and LMICs on Telehealth During COVID-19 Pandemic

The disruptive impact of COVID-19 has rapidly progressed the implementation and use of telehealth in Australian primary health care (PHC) as has occurred in other developed countries [26] (see Fig. 3). As cited in Tang & Reddy [27], the U.S. saw an increase of 154% in telehealth visits at the end of March 2022 compared to the same period in 2019 (see Table 5). A mixed-method study conducted in the UK and Canada postulates that although patients’ satisfaction with telehealth in musculoskeletal physical therapy is widely reported as high as face-to-face, widespread adoption of telehealth within physical therapy has been slow [13]. In Springfield, Massachusetts in the United States, there were 698 (39%) telehealth visits out of a total of 1788 prenatal visits. This shows that there was high adoption of telehealth by high-income countries like the U.S. during the COVID-19 pandemic [28] (see Table 5). In high-income countries, the COVID-19 pandemic forced health systems to quickly adapt to telehealth use for cancer treatment [29]. During this COVID-19 pandemic era, while specialties in some high-income countries like psychiatry, cardiology, and radiology are recording higher telehealth use, others like allergy-immunology, family medicine, and gastroenterology are recording lower telehealth use [3]. Based on responses from a study conducted in Mozambique, 69% of respondents were willing to use teleconsultation public health services for mild illness and review consultations during this COVID-19 pandemic era [30]. A study by Ranjbar et al., [31] in a low-and-middle-income country showed that out of a total of 523 nurses and midwives who participated in the study, 73.0% had a positive attitude toward telenursing and telehealth. Higher education was positively associated with the understanding of telehealth. Kazi et al., [32] make it known that telehealth was initially in its infancy in LMICs, however, the COVID-19 pandemic accelerated the demand for telehealth following social distancing protocols globally (see Table 5).

During the COVID-19 pandemic era, 78% of clinicians who took part in a study organised in the city of Makassar in Indonesia (LMIC) indicated their satisfaction with telehealth systems. 69% of participants indicated that telehealth allowed for quicker diagnosis and treatment [33] (see Table 6). In South Africa, telehealth is perceived as a mitigator of healthcare provider shortages, and remote access to healthcare services in this COVID-19 pandemic era [5]. The application of telehealth in the area of hypertension management in LMICs during this COVID-19 pandemic saw a significant reduction in blood pressure among hypertensive patients [6] (see Table 6). A study in Australia shows that telestroke has proven to be effective in the timely management of stroke conditions during the COVID-19 pandemic [34]. In resource-challenged areas in Brazil, teleconsultation was a strategic technological tool for patients to access quality healthcare in a COVID-19 pandemic era where social distancing is a new normal [35]. Implementation of telehealth in Nebraska has increased access to health services among rural residents and deeply impacted clinical practice. Clinicians in Nebraska plan to continue providing services via telehealth if policies and regulations are well-enacted post-COVID-19 [36]. The COVID-19 pandemic brought about the novel adoption of telehealth in the field of Orthopaedic Oncology in some high-income countries [37]. Evenski et al., [37] further indicated that 42% of study respondents rated tele-orthopedic services at 9.7 out of 10 (see Table 5).

**Table 6:** Application of telehealth in specialised services & advantages during COVID-19

High-Income Countries (HICs)		
Year	Author(s)	Key Findings
2021	Tsou et al., [34]	Telehealth is used in rural and remote emergency departments to effectively improve clinical care processes, and speed of care. Telestroke has been proven during the COVID-19 pandemic to be effective in the timely management of stroke conditions.
2022	Peixoto et al., [35]	In resource-challenged areas in Brazil, teleconsultation is a strategic technological tool for patients to access quality healthcare in a COVID-19 pandemic era where social distancing is a new normal.
2021	Freske & Malczyk, [36]	Implementation of telehealth in Nebraska has increased access to health services among rural residents and deeply impacted clinical practice. Clinicians in Nebraska plan to continue providing services via telehealth if policies and regulations are well-enacted post-COVID-19.
2021	Smith et al., [38]	Australia has implemented the Breastscreen Australia Remote Radiology Assessment Model (RRAM) to address the hurdle of inadequate access to a local radiological workforce in regional Australia. Majority of participants saw no difference between telehealth services and the onsite model.
2020	Evenski et al., [37]	The COVID-19 pandemic brought about the novel adoption of telehealth in the field of Orthopaedic Oncology, which is expected to positively impact healthcare access and compliance. 42% of participants in the Evenski et al., (2020) study rated tele-orthopedic services at 9.7 out of 10. This result is consistent with previous findings with telehealth in other specialties.
Low-and-Middle-Income Countries (LMICs)		
2020	Indria et al., [33]	During the COVID-19 pandemic era, 78% of clinicians who took part in a study organised in the city of Makassar in Indonesia indicated their satisfaction with telehealth systems. 69% of participants indicated that telehealth allowed for quicker diagnosis and treatment.
2022	Tahir et al., [5]	In South Africa, telehealth is perceived as a mitigator of healthcare provider shortages, and poor rural and remote access to healthcare services.
2021	Hoffer-Hawlik et al., [6]	The application of telehealth in the area of hypertension management in LMICs saw a significant reduction in blood pressure among hypertensive patients.

Barriers to using telehealth interventions in older adults were identified in some developed countries include knowledge gaps, lack of willingness to adopt new skills, and reluctance to technology use [39]. A scoping review conducted in the UK showed that there were no established uniform guidelines for telehealth implementation [40] (see Table 7). Although findings support the rapid adoption of telehealth in clinical care delivery in North America, the implementation of telehealth has faced critical challenges such as variations in state licensure requirements for telehealth; disparities in access to telehealth among disadvantaged populations; and lack of consistency among individual investigational review boards (IRBs) on telehealth studies [41]. The implementation of telehealth services in high-income countries often results in challenges stemming from the lack of attention to change management [42]. In a study conducted in rural Bangladesh (LMIC), exemplary barriers to telehealth adoption that were identified and confirmed (*p<*0.01) were: lack of organisational effectiveness, health staff motivation, patient satisfaction, and trustworthiness. In this same study, lack of Information Communication and Technology (ICT) infrastructures and allocation of resources were identified as indirect barriers [43]. In LMICs, telehealth system vulnerabilities may result in inappropriate access to patient information, medical device malfunction, or breakdown of health services that are provided, which may result in ethical and legal issues [44]. Existing telehealth services in Thailand are limited to only fundamental medical consultation services [45]. Lack of governance and stakeholder support, lack of effective logistical and clinical procedures, and patients’ ability to adapt to telehealth care are the barriers to the mass adoption of telehealth services in Pakistan (LMIC) [46] (see Table 7).

**Table 7:** Challenges of Telehealth Implementation During COVID-19

High-Income Countries (HICs)		
Year	Author(s)	Key Findings
2022	Zaman et al., [39]	Although telehealth interventions that were designed to help people self-manage chronic diseases demonstrated positive effects, barriers to using telehealth interventions in older adults were identified and some were: knowledge gaps, lack of willingness to adopt new skills, and reluctance to technology use.
2021	Leone et al., [40]	A scoping review conducted in the UK showed that there were no established uniform guidelines for telehealth implementation.
2021	Naito et al., [41]	Although findings support the rapid adoption of telehealth in clinical care delivery in North America, the implementation of telehealth has faced critical challenges such as variations in state licensure requirements for telehealth, disparities in access to telehealth among disadvantaged populations, lack of consistency among individual investigational review boards (IRBs) on telehealth studies.
2020	Kho et al., [42]	The implementation of telehealth services often result in challenges stemming from the lack of attention to change management.
Low-and-Middle-Income Countries (LMICs)		
2020	Zobair et al., [43]	In a study conducted in rural Bangladesh, exemplary barriers to telehealth adoption that were identified and confirmed (*p<*0.01) were; lack of organizational effectiveness, health staff motivation, patient satisfaction, and trustworthiness. Lack of Information Communication and Technology (ICT) infrastructures and allocation of resources were identified as indirect barriers.
2022	Haroon et al., [44]	Telehealth system vulnerabilities may result in inappropriate access to patient information, medical device malfunction, or breakdown of health services that are provided, which may result in ethical and legal issues.
2022	Poonsuph, [45]	Existing telehealth services in Thailand is limited to only fundamental medical consultation services.
2021	Mahdi et al., [46]	Lack of governance and stakeholder support, lack of effective logistical and clinical procedures, and patients’ ability to adapt to telehealth care are the barriers to the mass adoption of telehealth services in Pakistan.

## Conclusion

Before the COVID-19 pandemic, the use of telehealth was not common in both HICs and LMICs, and there were difficulties in its deployment. However, there were also effective applications of telehealth in specialized healthcare services, including telepsychiatric interventions and pediatric palliative care, as well as advantages for patients and healthcare workers in terms of cost savings. The impact of COVID-19 accelerated the adoption of telehealth at the facility level but not nationwide in both HICs and LMICs (see Table 2 and Table 5). The widespread implementation of telehealth is nevertheless hampered by knowledge gaps, a lack of guidelines, access inequities, and infrastructure issues, particularly in LMICs. The lack of national policies and regulations is making the adoption of telehealth at the national level challenging. The integration of telehealth into current healthcare systems and the reimbursement of telehealth services are two examples of policies that governments should develop to facilitate the use of telehealth in healthcare systems. Governments should set aside funds to upgrade the telehealth-related infrastructure and resources, including broadband internet access, telecommunication networks, and the purchase of telemedicine hardware. Healthcare professionals should be trained on how to use telehealth protocols and technology, including how to incorporate telehealth into their current practice and how to use electronic medical records. Governments should set up legal guidelines to safeguard patient information security, confidentiality, and privacy in telehealth systems. There were limited studies on telehealth with regards to LMICs. This research did not include articles from all HICs and LMICs. Inclusions were limited to studies written in English language, peer-reviewed journals, and studies published on or after 2012. This means that studies with equally good information which did not meet the inclusive criteria were excluded. Therefore, findings from this study may not be generalised. To increase the specificity of the research topic and methods, the study purposefully concentrated on the term of "telehealth" while omitting comparable concepts like "telemedicine" and "digital health". By ignoring the potential contributions of other similar concepts, this strategy may have hampered the generalizability of the findings, perhaps limiting the study's scope. Future research should focus on examining the subtleties and connections between various digital health topics. However, the study advances knowledge of the function of telehealth in global emergencies, and readers are urged to evaluate the results in light of the methodology and telehealth.

Primary quantitative and qualitative studies must be conducted to address challenges encountered during the pilot implementation of telehealth in both HICs and LMICs before and during the COVID-19 pandemic. Addressing these challenges will help countries adopt telehealth at a national level. LMICs must find innovative solutions to address peculiar challenges such as inadequate financial resources for mass-scale telehealth implementation, especially in sub-Saharan Africa.

## Data Availability

Pieces of Literature analysed during the current study are available online and can also be made available through the corresponding author upon request.
